# COVID-19 and Public Health Spending; Effects on the Economic Sustainability of the Spanish Private Healthcare System

**DOI:** 10.3390/ijerph20021585

**Published:** 2023-01-15

**Authors:** María del Carmen Valls Martínez, José Manuel Santos-Jaén, Rafael Félix Valls-Úbeda, Rafael Soriano Román

**Affiliations:** 1 Mediterranean Research Center on Economics and Sustainable Development, 04120 Almería, Spain; 2 Economics and Business Department, University of Almería, 04120 Almería, Spain; 3 Department of Accounting and Finance, University of Murcia, 30100 Murcia, Spain

**Keywords:** COVID-19, public health spending, economic sustainability, private healthcare system, profitability, corporate social responsibility

## Abstract

This research analyzes the influence of COVID-19 and public health spending policies on the economic sustainability of Spanish private hospitals. Moreover, it explores the relationship between public health spending policies and the spread of COVID-19 in Spain. Private health care is an understudied sector, yet it is fundamental to the health of citizens. Moreover, the economic causes linked to the spread of the pandemic have not yet been clearly established. Therefore, this work covers a gap in the literature. Private hospital profitability was analyzed by applying ordinary least squares and panel data regressions on financial and macroeconomic data for the period 2017–2020. The spread of COVID-19 was examined by means of cluster and component analysis. The results show that the COVID-19 pandemic negatively affected the economic sustainability of Spanish private hospitals, which was also influenced by public health spending. In turn, the spread of the pandemic was mainly related to population density, but was also influenced by public health spending and the gross domestic product of the region. Therefore, policymakers must consider that it is essential to make adequate investments in the healthcare system to cope with pandemics such as COVID-19. In addition, managers can see how corporate social responsibility is a valuable strategy for maintaining hospital profitability.

## 1. Introduction

The European social welfare system includes health care, pensions, education, and social services. However, the operating model is not uniform across countries [1]. In the continental system, which was established by Bismarck in Germany, the health services are supplied by private companies, although the state is responsible for their regulation. In contrast, in the Anglo-Saxon system, established in the United Kingdom by Sir William Beveridge, health care is provided by public hospitals, and private hospitals are complementary to public services. Spain employs what is known as the Mediterranean model, which is a symbiosis of the two previous models, such that health care is jointly provided by both public and private hospitals [2].

The entire Spanish population is covered by the public social security system, with three notable exceptions: justice administration staff, members of the armed forces, and all other government employees are covered by the Mutualidad General Judicial (MUGEJU), the Instituto Social de las Fuerzas Armadas (ISFAS), and the Mutualidad General de Funcionarios Civiles del Estado (MUFACE), respectively [3,4,5].

These may all choose to receive health care through either the public or the private systems, with the majority opting for private hospitals. Among the reasons given for choosing private health care is the possibility of directly visiting a specialist without the need for consulting the family doctor in advance; being able to choose a doctor or consult multiple specialists for a second opinion on a diagnosis or treatment; and avoiding long waiting lists.

On the other hand, the public hospital and healthcare system is insufficient to satisfy the needs of the population, resulting in long waiting lists. Patients wait an average of 99 days to be seen for a first consultation with a specialist, although there are notable differences between the different regions of the country. For example, while the average wait in País Vasco is 30 days, in Aragón it is 147 days. Similarly, the average waiting time for non-urgent surgery is 148 days, but with large differences depending on the place of residence. For example, the average waiting time in Asturias is 60 days, whereas in Castilla-La Mancha it is 286 days. Similarly, the percentage of patients waiting more than 6 months for non-urgent surgery was 26.9% in Spain. However, the differences between regions is notable; for example, in País Vasco it is 5.5%, whereas in Extremadura it is 40.2% [6]. Consequently, with the aim of reducing these waiting times, the public administration makes agreements with private hospitals to transfer certain consultations, diagnostic tests, and treatments to them. These are known as “conciertos”, or public-private contracts.

The Spanish public health system is organized into two levels of care. Primary care is the basic and initial level of care, characterized by its capacity to attend to frequent health problems and its easy accessibility. In fact, 15.5% of people are seen on the same day that they request an appointment, while 26.8% are seen the following day, and 50.6% have to wait an average of 5.8 days [6]. Primary care centers have family physicians and primary care nurses, who perform basic diagnostic tests. The second level of care is specialized care, which has the most complex diagnostic facilities, hospitals, specialist physicians, and specialized care nurses. This level is accessed on the advice of primary care physicians once their capabilities have been exceeded. Patients are treated at this second level until they are able to return to the first level. In 2019, primary care centers received 402.3 million consultations in Spain. For its part, specialized care carried out more than 100 million consultations, with 80% in public centers and 20% in private hospitals [6,7].

In short, private hospitals are used by public employees affiliated to the MUGEJU, ISFAS, and MUFACE mutual insurance companies who opt for private health care; by those patients who, through public-private contracts, are referred to the private system when it is impossible to be treated by the public system within a reasonable period; and by those citizens who, through private insurance or direct payment, decide to use private care as a complement to public healthcare. The total Spanish healthcare expenditure exceeds 9% of the GDP, and more than 30% of this expenditure corresponds to the private sector, which places Spain above the average for the main neighboring countries. Undoubtedly, private insurance provides savings to the national healthcare system, which was estimated at €1134 per person for 2016, contributing to its sustainability [8].

The main causes of death in Spain in 2020 were circulatory system diseases (24.3%), cancer (22.8%), and infectious diseases, including COVID-19 (16.4%) [9]. There were 71,208 more deaths in Spain in 2020 than in 2019, representing an increase of 17.9%, and 16.9% more than the median for the five years prior to 2020. The largest increases occurred for people over 70 years of age, with 20.5% more deaths than in 2019, and for nursing home residents, with an increase of 33.7%. These increases were most pronounced in the months of March and April, coinciding with the peak of the pandemic. Specifically, the mortality rate due to COVID-19 stood at 127.5 per 100,000 inhabitants. These deaths were concentrated in older age groups; specifically, about 90% of those who died from COVID-19 were 70 years of age or older [10].

The case of Spain is interesting because, despite having a healthcare system ranked among the best in the world, it was the European country with the highest COVID-19 incidence rate, and had one of the highest percentages of hospital admissions and deaths in 2020. This brutal impact was blamed on the mismanagement of the crisis by the authorities. Indeed, this was denounced by a group of scientists in the prestigious journal *The Lancet* [11], where they called for an independent evaluation of government management.

In addition, Spain was the European country with the highest economic impact of COVID-19 in terms of a fall in GDP in 2020, although there are significant differences between regions. The fall in GDP was greater in those areas more dependent on tourism, while it affected the regions that focus their activities more on the primary sector to a lesser extent [12].

Furthermore, the Spanish territory is divided into 17 regions, known as autonomous communities, with independent regional governments that have total authority over their own healthcare systems. Therefore, as each autonomous government has its own policies, there are actually 17 different healthcare systems in Spain [13,14].

As the level of public spending differs across the country, the quality of the public healthcare system also varies [15,16]. Thus, it is logical to think that in those regions with better public healthcare, the citizens will be less attracted to private healthcare. Therefore, the performance of private hospitals might be affected by the level of public health spending.

Similarly, the COVID-19 pandemic was heterogeneous across the Spanish territory. Some studies have pointed to mobility, immigration, and the economic structure of the different regions as determining factors [17]. Considering how the virus spread throughout the national territory and how, as previously mentioned, it influenced the economy, it would be interesting to analyze how it affected the economic sustainability of the private hospital sector and whether any relationship was observed with public health spending. In the context of this research, the economic sustainability of hospitals is identified with the achievement of profits in their management, in particular, with positive returns on assets.

The objective of this research is two-fold. First, we analyze the influence of the COVID-19 pandemic and public health spending policies on the economic sustainability of Spanish private hospitals. To do so, we analyzed the period 2017–2020, which includes the peak year of the pandemic, as well as the three previous years. Second, we explore the relationship between public spending policies and the spread of COVID-19 in Spain. None of these objectives have been previously studied. In fact, the private hospital sector has hardly been examined at all, despite its importance for the health of citizens and, consequently, the national economy. Therefore, the present work fills this gap in the literature.

The second section of this article describes the sample and variables used, as well as the different methodologies employed: ordinary least squares, panel data regression, hierarchical cluster analysis, and factor analysis. The third section shows the results of the study. Finally, the fourth section discusses the results and presents the main conclusions.

## 2. Materials and Methods

### 2.1. The Dataset

The data used in this study were all secondary data and were obtained from different sources. The financial and corporate data for the private hospitals analyzed were obtained from the AMADEUS (Bureau van Dijk) database. The search performed included those Spanish companies carrying out their activities under NACE (Nomenclature of Economic Activities in the European Union) code 8610, corresponding to Hospital Activities, and with operating revenues of more than €10 million and more than 50 employees during the period 2017–2020. These limits were established with the aim of eliminating from the study those small clinics specialized in a specific branch of medicine; e.g., ophthalmology clinics, dental clinics, etc. After refining the sample by eliminating those observations with missing or erroneous data, 269 observations remained (68 for 2017, 67 for 2018, 66 for 2019, and 68 for 2020). Hospitals from all Spanish regions were represented.

By analyzing the websites of the different hospitals, we determined whether they implemented corporate social responsibility policies.

The variables of public health expenditure per inhabitant and the frequency of COVID-19 cases by autonomous community were obtained from the website of the Spanish Ministry of Health. Finally, the average gross domestic products by autonomous community and population density were obtained from the website of the Spanish National Institute of Statistics, which provides publicly accessible macroeconomic variables.

### 2.2. Variable Description

Table 1 describes the variables used to study the relationship between the economic sustainability of private hospitals and both the COVID-19 pandemic and public health spending in the specific territory.

The proxy variable for economic sustainability was return on assets (ROA), widely used in profitability studies [18,19,20,21]. Appendix A shows the average annual ROA of the sample analyzed. It can be seen that, in 2020, profitability sharply fell (2.4) with respect to the average values obtained in the previous three years (5.01, 5.76, and 4.95 for 2017, 2018, and 2019, respectively).

To determine the influence of the COVID-19 pandemic, a dummy variable was created (COV) that took the value 1 if the year corresponded to the peak year of the pandemic, i.e., 2020, and 0 otherwise, with an expected negative and significant relationship with economic profitability.

In Spain, the public health system offers universal coverage for all citizens. However, the private healthcare system is also highly developed and is used by a significant part of the population, mainly through private health insurance, as this allows them to choose which specialist they see and to receive medical attention more quickly by avoiding the usual long waiting lists of the public health system. In those autonomous communities with better public healthcare systems, i.e., those with more beds available, more diagnostic instruments (computerized axial tomography equipment, magnetic resonance imaging, etc.), and more specialist doctors [1,15,22], it is logical to think that citizens will have less need to resort to the private system as a complementary service. Moreover, given the cost of private health insurance, only those with higher incomes can have access to this service. Therefore, two macroeconomic variables were included to determine the economic sustainability of private hospitals: the average public health expenditure per inhabitant (EXP) and the per capita gross domestic product (GDP) corresponding to the autonomous community in which the hospital is located. In the first case, the expected relationship with the economic sustainability of hospitals is negative, while in the second case a positive relationship is expected.

A large amount of the literature has studied the effects of corporate social responsibility (CSR) policies on a company’s financial returns. Most of the empirical research concludes that the influence is positive [23,24,25,26,27], although some studies have found either a negative relationship or no relationship at all between corporate social responsibility and economic performance [28].

Gender diversity in the boardroom is a controversial issue in both legislation and academic literature, and is, therefore, a hot topic for research. Traditionally, boards of directors were occupied by men, but increasingly more women have been incorporated in recent years into top management positions, and the influence of gender on profitability is now frequently studied from a theoretical and, above all, empirical perspective. The results so far have not been conclusive. While a large number of studies have found that a greater presence of women has a positive and significant influence on profitability [29,30,31], other studies have found no such relationship [32,33].

Traditional profitability studies use company size [34,35,36], asset turnover [37,38], and level of indebtedness [39,40,41,42] as determinants of ROA. Total assets, operating income, and number of employees have traditionally been used as proxies for company size. In this research, the number of employees (EMP) was considered to be more representative of hospital size. Larger companies, which can take advantage of economies of scale due to their size, are expected to have a higher ROA. Asset turnover (TUR) is also usually directly related to profitability. However, indebtedness (IND) usually has an inverse relationship with profitability, although a certain degree of leverage is considered positive for the company. In other words, the use of borrowed funds, provided this is not excessive, can favor good financial performance.

Hospital age (AGE) may be an influential variable in ROA [43,44,45]. Older hospitals may hold prestige among the population, while newer hospitals may have the latest technology for diagnosis and treatment.

Legal form (LEG) is a variable that has been used in previous studies on profitability [46]. In this research, we have considered both public and private limited companies.

Finally, to study the relationship between public health spending and the frequency of COVID-19 cases, two additional variables were used for each autonomous community: the average number of COVID-19 cases per 100,000 inhabitants in 2020, and population density, i.e., the average number of inhabitants per km². The population density variable was chosen as a socio-environmental variable because the transmission of the virus is by close proximity to other people already infected.

### 2.3. Statistical Analysis

Different methodologies were used for data analysis, depending on the intended objective. To determine whether, and to what degree, the economic sustainability of private hospitals was affected by the COVID-19 pandemic and by public health spending, we conducted a descriptive analysis of the continuous variables involved in the study and their bivariate correlations. For the dichotomous variables, mean and ANOVA tests related to the ROA were performed.

Then, a multivariate analysis was applied. In Model 1, we tested a multiple linear regression using ordinary least squares (OLS):

(1) $$\begin{array}{c} ROA_{j}=\beta_{0}+\beta_{1}COV_{j}+\beta_{2}EXP_{j}+\beta_{3}GDP_{j}+\beta_{4}CSR_{j}+\beta_{5}BGD_{j}+\beta_{6}EMP_{j}+\\ +\beta_{7}TUR_{j}+\beta_{8}IND_{j}+\beta_{9}AGE_{j}+\beta_{10}LEG_{j}+\xi_{j} \end{array}$$

where *j* corresponds to the hospital and $\xi_{i}$ is the random disturbance.

In Model 2, the one-period lagged dependent variable was used as a regressor in order to address possible endogeneity problems, in accordance with previous literature [25,47,48]:

(2) $$\begin{array}{c} ROA_{j}=\beta_{0}+\beta_{1}ROA_{1\mathrm{lag},j}+\beta_{2}COV_{j}+\beta_{3}EXP_{j}+\beta_{4}GDP_{j}+\beta_{5}CSR_{j}+\beta_{6}BGD_{j}+\\ +\beta_{7}EMP_{j}+\beta_{8}TUR_{j}+\beta_{9}IND_{j}+\beta_{10}AGE_{j}+\beta_{11}LEG_{j}+\xi_{j} \end{array}$$

Finally, to control for problems caused by omitted variables, panel data combining longitudinal and cross-sectional data were considered. Thus, fixed and random effects were applied in Models 3 and 4, respectively. The fixed effects model is appropriate when unobservable heterogeneity among hospitals is correlated with the regressors. Otherwise, the random effects model is preferable. The appropriate choice between the two models is determined by applying the Hausman test [49], such that, if the *p*-value is less than 0.05, fixed effects will be preferred.

(3) $$\begin{array}{c} ROA_{j,t}=\beta_{0}+\beta_{1}COV_{j,t}+\beta_{2}EXP_{j,t}+\beta_{3}GDP_{j,t}+\beta_{4}CSR_{j,t}+\beta_{5}BGD_{j,t}+\beta_{6}EMP_{j,t}+\\ +\beta_{7}TUR_{j,t}+\beta_{8}IND_{j,t}+\beta_{9}AGE_{j,t}+\beta_{10}LEG_{j,t}+\xi_{j,t} \end{array}$$

where *t* corresponds to the year.

The Akaike (AIC) and Bayesian Information Criteria (BIC) were used to select the model that best fits the data of the sample analyzed, as lower values are identified with the best model [50,51].

Once the best model had been selected, different tests were applied to check its validity. The Jarque-Bera test [52], based on the skewness and kurtosis of the distribution, verifies the normality of the residuals if *p*-value > 0.05. Similarly, the Breusch-Pagan test [53] establishes the homoscedasticity of the residuals, and the Ramsey test [54] indicates that there are no relevant omitted variables. Moreover, the *F*-test shows the validity of the overall model, and the goodness of fit coefficient *R*^2^ shows the proportion of explained variance.

To test the robustness of the final model, a robust regression and an estimation with the winsorized variables were performed. In this way, the stability of the coefficients was tested. Regression analyses and the rest of the above tests were performed with STATA v.16 software (StataCorp, College Station, TX, USA).

To analyze the relationship between public health expenditure, GDP, the population density of each autonomous community, and the number of COVID-19 cases in 2020, an agglomerative hierarchical cluster analysis based on Ward’s minimum variance criterion was conducted. Initially, all clusters have a single element. At each step, the two clusters with the minimum distance between them are merged, such that, at each step, the pair of clusters that leads to the minimum increase in the total cluster variance resulting after merging is found. In the end, a single cluster includes all individuals in the sample [55,56].

By means of a factor analysis, the variables under study were reduced to only two dimensions, called factors. Based on these, the clusters were graphically represented, in order to obtain a simpler and more visual relationship between the different groups [57,58,59]. Cluster and factor analyses were performed with SPSS v.27 software (IBM, Armonk NY, USA).

## 3. Results

### 3.1. Regression Analysis

#### 3.1.1. Descriptive Statistics and Bivariate Relationships

Table 2 presents the descriptive statistics of the continuous variables (note that some variables are given in terms of logarithm, as can be seen in Table 1, as they are considered in this format in the regression analysis). The average economic profitability of Spanish hospitals during the period 2017–2020 was 4.52%, but with a large variance, ranging from a minimum of −11.73% to a maximum of 24.65%.

Regarding public health expenditure per autonomous community, the national average is 1505.45 euros per inhabitant. However, the difference between the regions of the country is remarkable since, while some areas devote 1199.24 euros per inhabitant to public health spending, others invest 1918.90 euros, which is 60% higher.

GDP per capita also significantly varies between autonomous communities, ranging from 17,448 to 36,049 euros. In other words, GDP in the richest regions is 106.61% higher than in the poorest areas of the country.

Gender diversity on hospital boards stands at an average of 21.17%, ranging from those companies with a total absence of women to those where they represent 60% of the board of directors.

The average level of indebtedness of Spanish private hospitals stands at 52.64%, but the distribution is highly uneven, with the figures ranging from only 5.37% to 99.18% of the total funds.

Other variables include the number of employees, asset turnover, and company age, with average values of 532 employees, 1.31 times, and 30.53 years, respectively.

Regarding dichotomous variables, most private hospitals in Spain have the legal form of a public limited company (60.59%), and that a large majority apply corporate social responsibility policies (71.75%). In addition, 25% of the observations obviously correspond to the peak period of the COVID-19 pandemic, i.e., 2020, since the study period is 2017–2020.

Appendix B provides the Pearson correlations between the continuous variables. It can be observed that public health expenditure (EXP) is negatively and significantly correlated with the profitability of private hospitals (ROA). Other variables that also inversely move to profitability are indebtedness, company age, and the percentage of women on the board of directors. The correlations between the different variables are not high, which indicates that, a priori, there should be no problems of collinearity in the regression models.

Appendix C shows the difference in means of the ROA variable according to each of the dichotomous explanatory variables and the ANOVA test. It can be seen that such differences are significant for all the variables, which indicates the convenience of including them in the regression model. Profitability during the peak year of the pandemic was significantly lower in those companies that do not apply corporate social responsibility measures and in hospitals with the legal form of a public limited company.

#### 3.1.2. Multivariate Analysis

Table 3 summarizes the results of the regression analysis. Looking at the AIC and BIC values, Model 2 outperformed Models 1 and 3. In turn, Model 3, according to the Hausman test (*p*-value > 0.05), was discarded in favor of the random effects Model 4. The COV variable, which reflects the effect of the COVID-19 pandemic on the ROA of hospitals, showed a negative and significant effect. Therefore, it can be stated that the pandemic led to a reduction in the economic profitability of Spanish private hospitals.

Public health spending in the autonomous communities showed a negative relationship with ROA, which was significant in Model 4.

The percentage of women on the board of directors also showed, in Models 1 and 2, a negative and significant influence on ROA. However, corporate social responsibility did show a positive influence on ROA in both models, but only significantly in Model 1. Both variables could not be tested in the panel data models, since they were considered to be stable data for the period analyzed.

Another variable that is positively related to economic profitability is asset turnover. In contrast, level of indebtedness is generally considered to be inversely related, although in Model 2 it has a positive sign, albeit non-significant.

Model 2 succeeds in explaining 61.57% of the ROA. We can affirm that there are no collinearity problems between the regressors, since the highest value of the VIF (variance inflation factor) is 1.4. According to the Jarque-Bera test, the residuals follow a normal pattern (*p*-value = 0.1481). Similarly, according to the Breush-Pagan test, they show homoscedasticity (chi^2^ = 0.78 and *p*-value = 0.3781). Furthermore, based on the Ramsey test, we can affirm that there are no relevant omitted variables (*F* = 1.21 and *p*-value = 0.3073).

#### 3.1.3. Robustness Checks

In order to test the robustness of the results, Model 2 was selected, since it returned the lowest values in the AIC and BIC criteria. The appendix shows (Appendix D) the results corresponding to the estimation with the robust option and the estimation with the winsorized variables at the 1% level. It can be seen that, indeed, the model remains stable, since the coefficients and their significance show minimal variance.

### 3.2. Cluster Analysis

**Figure 1:**
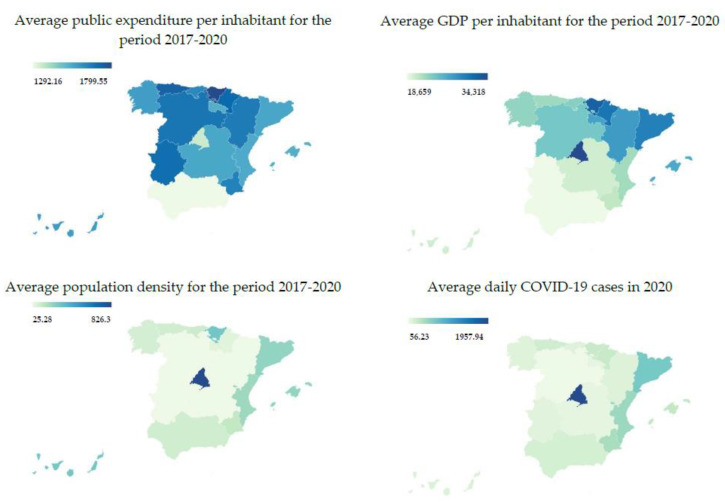
Data by autonomous community.

Table 4 collects the data corresponding to the four variables used for the hierarchical cluster analysis of the Spanish regions. These data are represented by choropleth maps in Figure 1 (the maps were made with the Datawrapper tool).

A priori, there is a certain correspondence between population density and COVID-19 infection rates, which seems logical considering that contagion occurs through physical proximity between individuals. The same occurs, although to a lesser extent, between the economic variables of public health expenditure and GDP, i.e., in those areas where GDP is greater and, therefore, the inhabitants are wealthier, public health spending tends to be higher. However, it can be seen that the relationship between COVID-19 infection rates and public health expenditure or GDP is not as obvious in the case of Spain.

**Figure 2:**
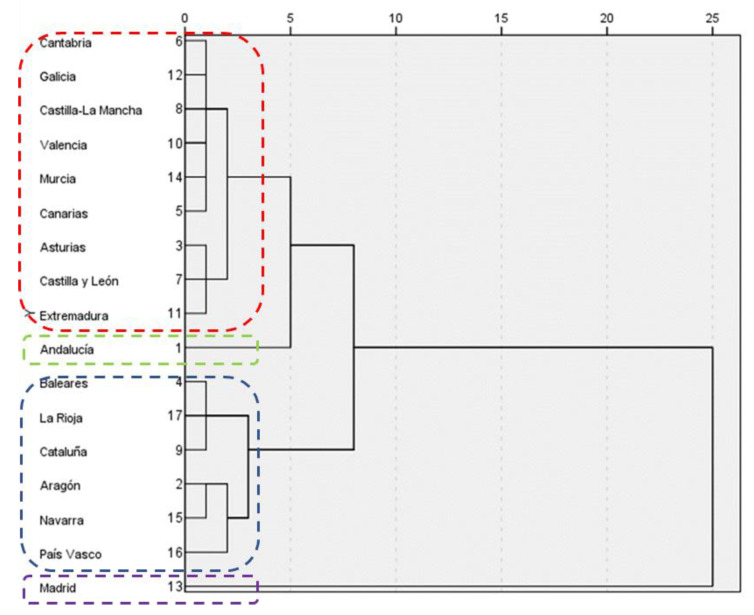
Cluster analysis by distance based on Ward’s method.

Figure 2 shows the hierarchical cluster analysis derived from the analyzed data. Four clearly differentiated clusters can be considered. Cluster 2, which is highlighted in red, includes nine regions: Cantabria, Galicia, Castilla-La Mancha, Valencia, Murcia, Islas Canarias, Asturias, Castilla-León, and Extremadura. Cluster 3, which is marked in blue, includes the regions of Islas Baleares, La Rioja, Cataluña, Aragón, Navarra, and the País Vasco. Andalucía (in green) and Madrid (in purple), clusters 1 and 4, respectively, are isolated cases that cannot be compared with the rest of Spain.

**Figure 3:**
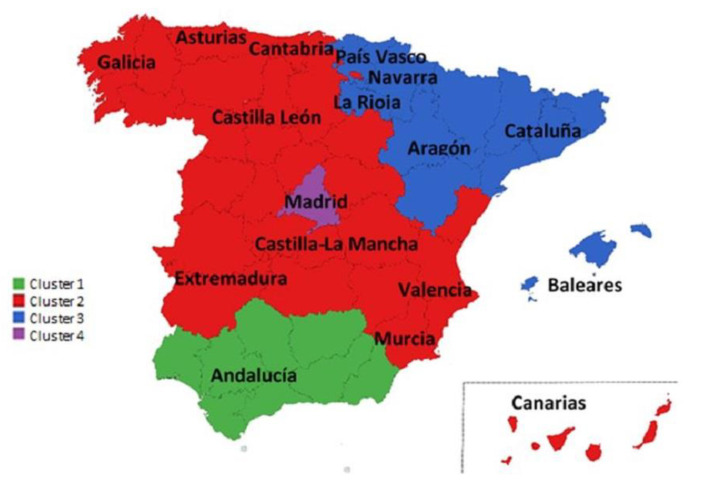
Cluster map.

Figure 3 shows their geographic distribution on the map of the country.

Appendix E shows the means of each variable by cluster. It can be seen that, except for the community of Madrid (cluster 4), public health spending is higher in those areas with a higher GDP. Furthermore, COVID-19 infection rates are directly related to population density. However, the relationship between expenditure and COVID-19 is not clearly defined.

### 3.3. Factor Analysis

The reduction of the variables analyzed to only two dimensions, through factor analysis, is shown in Table 5. Factor 1 has significantly higher values for the COVID-19 and population density variables, so we can call this factor “risk”. Factor 2, on the other hand, has a significantly higher value for public health expenditure, which is why we will refer to it as “budget”. Both factors have similar GDP values. These two dimensions explain 90.379% of the variance. Bartlett’s test yields a *p*-value of 0.000 < 0.05, and the Kaiser-Meyer-Olkin measure of sampling adequacy is 0.641. Therefore, the factor analysis is correct.

Appendix F shows the graphical representation of the autonomous communities according to factors 1 (risk) and 2 (budget). It can be seen that Madrid, with a low budget, shows an enormously high risk. Andalucía, with an excessively low budget, shows a medium risk. Both are clearly exceptions. With respect to the other Spanish autonomous communities, those included in cluster 3 have, on average, a lower budget and a somewhat lower risk than the regions in cluster 2.

To better understand this relationship between spending, GDP, population density, and COVID-19, we performed a regression analysis, obtaining the results shown in Table 6. 

It can be seen that COVID-19 infection rates are significantly and negatively correlated with public health expenditure, but positively correlated with GDP and population density. The individual regressions of each variable confirm these relationships. However, the joint regression indicates that only population density is significant for pandemic intensity.

## 4. Discussion

This article analyzes the influence of the COVID-19 pandemic and public health spending policies on the economic sustainability of Spanish private hospitals, and also explores the relationship between public spending policies and the spread of COVID-19 in Spain.

For this purpose, economic-financial data, the CSR practices of private hospitals, and macroeconomic information for the period 2017–2020 were analyzed. At this point, it should be noted that the different autonomous communities that integrate Spain show significant differences in public healthcare spending, per capita income levels, and gender diversity on hospital boards of directors.

Moreover, this research has tried to clarify the reasons for the significant disparities in COVID-19 cases among the autonomous communities that comprise Spain. To this end, a cluster analysis was performed due to the existing differences between the autonomous communities in terms of health system management, per capita income, and population density. This analysis includes the number of COVID-19 cases for the year 2020. In addition, through a component analysis, the clusters obtained were represented according to two factors: the risk of contracting COVID-19 and budget level.

The results show the effect of COVID-19 and public health spending policies on hospital sustainability. As for hospital profitability, a negative influence of public health spending was found, i.e., the greater the resources available to a public health system, the greater the satisfaction of its users [16] and, therefore, the less likely they will be to resort to the private system. In addition, a negative relationship was also found between indebtedness and the percentage of women on the board of directors. The latter contradicts the results of previous studies that affirmed the existence of a positive relationship [29,30,31] and those that denied the existence of a significant relationship [32,33]. However, the relationship between corporate social responsibility and profitability was positive.

Similarly, the findings show that, in 2020, the COVID-19 pandemic caused a sharp drop in hospital profitability. This was mainly due to the increase in costs, especially of personnel and materials, that hospitals had to bear to cope with the early stages of the pandemic. The empirical analysis performed also confirms the findings of previous studies regarding the positive effect of corporate social responsibility and corporate performance [23,24,25,26,27].

With respect to the second objective of this research, the cluster analysis shows that population density is strongly related to COVID-19 incidence rates, which is logical and to be expected given that this disease is spread by physical proximity between people.

Although the influence of population density is so high that it absorbs all other factors, it has also been shown that the higher the per capita income of the inhabitants, the higher the number of COVID-19 cases, which is in line with previous research [17]. This finding could be explained by the fact that more interaction between people is necessary in order to carry out greater productive activity.

Finally, the component analysis confirms the previous results and shows that, in those regions with higher public health spending, the risk of contracting COVID-19 was lower. This is due to the greater preventive capacity of health systems with greater resources. In other words, a higher number of primary care physicians and personnel in charge of case tracing enabled these regions to break the chain of transmission of the virus to a greater extent.

This work is not without its limitations, which could be explored in future lines of research. The present study was conducted only in Spain, so the results may not be extrapolated to other geographical areas. It would also be interesting to replicate this study in other health systems. Furthermore, hospital sustainability was measured only in terms of profitability. Therefore, future research could delve deeper into the social and environmental aspects of hospitals. Finally, this research only analyzed the number of COVID-19 cases. Future studies could analyze the effects of the pandemic, i.e., hospitalizations and the associated healthcare costs, as well as mortality, with its enormous associated social cost.

## 5. Conclusions

The sustainability of private hospitals is influenced by public health spending policies and by the effects of COVID-19. In addition, it has been shown that COVID-19 incidence rates were mainly due to population density. However, the spread of the virus was also affected by increased productive activity related to a higher GDP, and was slowed down by the resources provided through higher public health spending.

This research contributes two essential values to the literature by studying the sustainability of the private hospital sector and by analyzing the relationship between public health expenditure and the spread of the COVID-19 virus.

From a practical perspective, the findings have practical implications for policymakers and managers. For the former, the results show that it is essential to make adequate investments in the healthcare system to cope with pandemics such as COVID-19. Furthermore, for the latter, CSR has proven to be a valuable strategy for maintaining hospital profitability even during difficult times, such as the COVID-19 pandemic.

## Figures and Tables

**Table 1:** Variable description.

	Abbreviation	Variable	Definition
Dependent variable	ROA	Return on Assets	Earnings before interest and taxes divided by total assets.
Independent variables	COV	COVID-19	Dummy variable, which takes the value 1 in 2020 and 0, otherwise.
	EXP	Public expenditure	Logarithm of the public health expenditure per inhabitant in the autonomous community where the hospital is located.
Control variables	GDP	Gross domestic product	Logarithm of the gross domestic product per capita in the autonomous community where the hospital is located.
	CSR	Corporate Social Responsibility	Dummy variable, which takes the value 1 if the hospital informs about corporate social responsibility policies and 0, otherwise.
	BGD	Board gender diversity	Percentage of women on board of directors.
	EMP	Size	Logarithm of the number of employees.
	TUR	Asset turnover	Operating income divided by total assets.
	IND	Indebtedness	Liabilities divided by total assets.
	AGE	Age	Age of the hospital in years.
	LEG	Legal form	Dummy variable, which takes the value 1 for public limited companies (hospitals) and 0 for private limited companies (hospitals)

**Table 2:** Descriptive statistics of the continuous variables.

Variable	Mean	SD *	Minimum	Maximum
ROA	4.52412	6.69511	−11.72604	24.6529
EXP	7.31093	0.10926	7.08944	7.55951
GDP	10.15304	0.22366	9.76698	10.49263
BGD	0.21166	0.16199	0.00000	0.60000
EMP	5.96000	0.74774	4.34380	8.00470
TUR	1.31038	0.77716	0.33626	4.36441
IND	52.63843	24.02508	5.37252	99.17978
AGE	30.53147	20.97856	1.29315	86.54521

Number of observations: 269. * Standard deviation.

**Table 3:** Regression analysis.

Variable	OLS Model 1	OLS Model 2	Fixed Effect Model 3	Random Effect Model 4
Intercept	32.29106 (0.325)	0.224872 (0.993)	−144.8449 (0.327)	60.444250 (0.157)
ROA (1lag)		0.747980 *** (0.000)		
COV	−2.055239 ** (0.024)	−2.019761 *** (0.002)	1.104898 (0.624)	−1.937761 *** (0.004)
EXP	−5.692013 (0.130)	−2.599006 (0.366)	−10.70588 (0.110)	−8.118934 * (0.064)
GDP	1.602603 (0.384)	2.031580 (0.156)	22.44289 (0.129)	0.436807 (0.885)
CSR	1.743331 ** (0.046)	0.498630 (0.466)		
BGD	−9.309939 *** (0.000)	−3.273693 * (0.088)		
EMP	0.236134 (0.668)	−0.400910 (0.353)	1.882596 (0.524)	0.183161 (0.840)
TUR	1.190069 ** (0.021)	0.643189 (0.122)	3.815806 ** (0.013)	1.601277 ** (0.040)
IND	−0.046549 *** (0.006)	0.021153 (0.118)	−0.053214 (0.249)	−0.053979 ** (0.032)
AGE	−0.009181 (0.662)	0.0020243 (0.904)	−0.456028 (0.433)	−0.032926 (0.329)
LEG	−2.345456 *** (0.009)	0.038242 (0.956)		
Adjusted *R*^2^	0.1521	0.6157	0.6817	
*F*-statistic	5.81 *** (0.0000)	29.25 *** (0.0000)	5.84 *** (0.0000)	
Wald Chi2				40.69 *** (0.0000)
Hausman test			6.70 (0.4613)	
Observations	269	195	269	269
AIC	1752.717	1111.556	1402.289	
BIC	1792.259	1150.832	1431.046	

***, **, and * indicate less than 1% significance level, less than 5%, and less than 10%, respectively. OLS: Ordinary Least Square regression. AIC and BIC: Akaike and Bayesian, respectively, Information Criteria.

**Table 4:** Average by autonomous communities of the variables used in the cluster analysis for the period 2017–2020.

Autonomous Community	Public Expenditure per Inhabitant (€)	Gross Domestic Product per Inhabitant (€)	COVID-19 Cases in 2020 per 100,000 Inhabitants	Population Density by km^2^
Andalucía	1292.16	18,717.50	205.28	96.29
Aragón	1666.86	27,594.75	161.94	27.70
Asturias	1737.20	22,250.25	232.91	96.42
Islas Baleares	1529.13	26,380.00	306.77	237.72
Islas Canarias	1576.87	20,053.50	169.48	295.06
Cantabria	1629.31	23,217.25	150.82	109.29
Castilla-León	1681.79	23,874.00	56.23	25.54
Castilla-La Mancha	1549.59	20,065.50	114.72	25.63
Cataluña	1550.60	29,807.25	681.17	235.36
Valencia	1539.71	22,080.00	506.66	215.09
Extremadura	1702.33	18,659.00	140.78	25.58
Galicia	1576.11	22,803.75	151.68	91.29
Madrid	1344.70	34,318.00	1957.94	826.30
Murcia	1647.99	20,795.50	438.16	131.63
Navarra	1714.47	30,716.25	276.81	62.48
País Vasco	1799.55	32,396.50	269.27	301.05
La Rioja	1528.26	26,976.75	109.74	62.22

**Table 5:** Factor matrix.

Variable	Factor 1	Factor 2
COVID-19	0.961	−0.025
Public expenditure	−0.540	0.788
Gross domestic product	0.665	0.658
Population density	0.950	0.012
Total variance explained	90.379%	

**Table 6:** Determinants in the frequency of COVID-19 cases.

PANEL A. Pearson Correlations				
	COVID	Public Expenditure		GDP
Public expenditure	−0.4831 ** (0.0495)			
GDP	0.5602 ** (0.0194)	0.0555 (0.8326)		
Population density	0.9112 *** (0.0000)	−0.4365 * (0.0799)		0.5576 ** (0.0200)
**PANEL B**. COVID-19 Regression Analysis				
	Model a	Model b	Model c	Model d
Public expenditure	−1.638065 ** (0.049)			−0.538591 (0.242)
GDP		0.051108 ** (0.019)		0.013179 (0.323)
Population density			2.088758 *** (0.000)	1.745240 *** (0.000)
Intercept	2956.895 ** (0.029)	−915.9493 * (0.082)	−3.006629 (0.962)	586.2097 (0.394)
Adjusted *R*²	0.1822	0.2680	0.8190	0.8171
*F*-statistics	4.57 ** (0.0495)	6.86 ** (0.0194)	73.39 *** (0.0000)	24.83 *** (0.0000)

***, ** and * indicate less than 1% significance level, less than 5%, and less than 10%, respectively. Number of observations: 17.

## Data Availability

The data that support the findings of this study are available from the corresponding author upon request.

## Appendix A. Average Annual ROA

**Source**: Own elaboration.

## Appendix B. Pearson Correlations between the Continuous Variables

	**ROA**	**EMP**	**TUR**	**IND**	**AGE**	**EXP**	**GDP**
**EMP**	−0.0293 (0.6326)						
**TUR**	0.1283 ** (0.0355)	−0.1879 *** 0.0020					
**IND**	−0.1465 ** (0.0162)	0.1263 ** (0.0384)	0.0225 (0.7136)				
**AGE**	−0.1261 ** (0.0387)	−0.1271 ** (0.0373)	−0.1313 ** (0.0313)	−0.2258 *** (0.0002)			
**EXP**	−0.1592 *** (0.0089)	−0.1642 *** (0.0070)	0.0225 (0.7134)	−0.0745 (0.2234)	0.2184 *** (0.0003)		
**GDP**	0.0001 (0.9985)	0.1035 * (0.0902)	0.1044 * (0.0875)	0.1836 ** (0.0025)	−0.0419 (0.4937)	−0.0347 (0.5714)	
**BGD**	−0.2717 *** (0.0000)	0.1229 ** (0.0440)	−0.0407 (0.5061)	0.0892 (0.1447)	0.1108 * (0.0695)	0.1181 * (0.0531)	0.0658 (0.2819)
***, ** and * indicate less than 1% significance level, less than 5%, and less than 10%, respectively. Number of observations: 269.							

## Appendix C. Differences of Means in the Value of the Explanatory Dummy Variables and ANOVA Test

	**Difference of Means Test (*t*-Test)**			**ANOVA Test**	
**Variable**	**Mean Group 0**	**Mean Group 1**	**Difference^(+)^**	***F* ^(+)^**	**Adjust *R*^2^**
COV	5.242972	2.399293	2.843679 *** (0.0023)	9.46 *** (0.0023)	0.0306
CSR	3.252319	5.024938	−1.772618 * (0.0504)	3.86 * (0.0504)	0.0106
LEG	5.681170	3.771688	1.909482 ** (0.0220)	5.31 ** (0.0220)	0.0158
***, ** and * indicate less than 1% significance level, less than 5%, and less than 10%, respectively. Number of observations: 269.					

## Appendix D. Robustness Checks

**Variable**	**OLS (Model 2)**	**OLS Robust**	**OLS Winsorized ^†^**
Intercept	0.224872 (0.993)	0.224872 (0.993)	−0.554904 (0.983)
ROA (1lag)	0.747980 *** (0.000)	0.747980 *** (0.000)	0.746386 *** (0.000)
COV	−2.019761 *** (0.002)	−2.019761 *** (0.007)	−1.997233 *** (0.003)
EXP	−2.599006 (0.366)	−2.599006 (0.330)	−2.500266 (0.382)
GDP	2.031580 (0.156)	2.031580 (0.165)	2.022805 (0.155)
CSR	0.498630 (0.466)	0.498630 (0.451)	0.498439 (0.464)
BGD	−3.273693 * (0.088)	−3.273693 * (0.090)	−3.345440 (0.464)
EMP	−0.400910 (0.353)	−0.400910 (0.418)	−0.382390 (0.375)
TUR	0.643189 (0.122)	0.643189 (0.151)	0.650638 (0.120)
IND	0.021153 (0.118)	0.021153 * (0.098)	0.021462 (0.111)
AGE	0.0020243 (0.904)	0.0020243 (0.912)	0.002925 (0.861)
LEG	0.038242 (0.956)	0.038242 (0.954)	0.041600 (0.952)
Adjusted *R*^2^	0.6157	0.6374	0.6154
*F*-statistic	29.25 *** (0.0000)	29.84 *** (0.0000)	29.22 *** (0.0000)
Observations	195	195	195
AIC	1111.556	1111.556	1109.865
BIC	1150.832	1150.832	1149.141
*** and * indicate less than 1% significance level and less than 10%, respectively. ^†^ winsorized variables at the 0.01 level. OLS: Ordinary Least Square regression. AIC and BIC: Akaike and Bayesian, respectively, Information Criteria.			

## Appendix E. Average of Variables by Cluster for the Period 2017–2020

**Cluster**	**Public Expenditure per Inhabitant**	**Gross Domestic Product per Inhabitant**	**COVID-19 Cases in 2020**	**Population Density**
1	1292.16	18,717.50	205.28	96.29
2	1631.48	28,978.58	300.95	154.42
3	1626.77	21,533.19	217.94	112.72
4	1344.69	34,318.00	1957.94	826.30
